# Retrospective Analysis of Leishmaniasis in Sicily (Italy) from 2013 to 2021: One-Health Impact and Future Control Strategies

**DOI:** 10.3390/microorganisms10091704

**Published:** 2022-08-24

**Authors:** Federica Bruno, Fabrizio Vitale, Francesco La Russa, Stefano Reale, Gerald F. Späth, Eugenia Oliveri, Valeria Gargano, Viviana Valenza, Flavia Facciponte, Susanna Giardina, Giorgio Marino, Antonella Galante, Germano Castelli

**Affiliations:** 1 National Reference Center for Leishmaniasis (C.Re.Na.L.), Istituto Zooprofilattico Sperimentale della Sicilia, 90129 Palermo, Italy; 2 Laboratorio di Entomologia, Istituto Zooprofilattico Sperimentale della Sicilia, 90129 Palermo, Italy; 3 Tecnologie Diagnostiche Innovative, Istituto Zooprofilattico Sperimentale della Sicilia, 90129 Palermo, Italy; 4 Unité de Parasitologie Moléculaire et Signalisation, Institut Pasteur, Université Paris Cité, INSERM U1201, F-75015 Paris, France; 5 Istituto Zooprofilattico Sperimentale della Sicilia, 90129 Palermo, Italy

**Keywords:** Leishmaniasis, human Leishmaniasis, feline Leishmaniasis, canine Leishmaniasis, One-Health, sandflies, Leishmaniasis epidemiology, diagnosis

## Abstract

Leishmaniasis is an important vector-borne disease that represents a serious public health problem, including in Sicily (Italy), which is considered an endemic area. We collected canine, feline and human data from 2013 to 2021 in Sicily, while entomological surveys were conducted only in 2013 and 2021. Overall, 23,794/74,349 (34.4%) of dogs and 274/4774 (11.8%) of cats were positive in one or more diagnostic tests. A total of 467 cases of human Leishmaniasis were reported, with 71% showing cutaneous and 29% visceral involvement. The provinces with the largest number of patients were Agrigento (45.4%) and Palermo (37%). In 2013, *Phlebotomus perfiliewi* was the dominant sandfly species in Sicily (68.7%), followed by *Phlebotomus perniciosus* (17.2%) and *Sergentomya minuta* (14%). In 2021, *Phlebotomus* *perfiliewi* was confirmed as the most common species (61.6%), followed by *Phlebotomus* *perniciosus* (33.1%) and *Sergentomya* *minuta* (4.7%). Of particular interest was the identification of *Phlebotomus papatasi* (0.41%) in Agrigento. Our retrospective study can inform health authorities for the development of appropriate screening, treatment and control strategies to reduce Leishmania incidence rate. This study examined the present state of Leishmaniasis control, surveillance, and prevention in Sicily, but also highlighted deficiencies that could be addressed through the application of One-Health principles.

## 1. Introduction

Leishmaniasis is a vector-borne disease transmitted by obligate intracellular protozoa of the genus Leishmania [1,2,3,4,5]. Leishmania parasites cause a broad range of clinical manifestations, such as cutaneous (CL), mucocutaneous (MCL), visceral (VL), post-kala-azar dermal (PKDL), and diffuse cutaneous Leishmaniasis (DCL), depending on the Leishmania species and the immune response of the patient [4]. Over one billion people are at risk for infection and live in areas endemic for Leishmaniasis, estimating 30,000 new VL cases of and more than 1 million new CL cases each year [5,6,7]. Over 90% of annual VL incidences occur in six countries: Bangladesh, Sudan, India, Nepal, Ethiopia, Brazil and South Sudan [3,6,8,9,10,11]. East Africa is the second largest center of VL after the Indian continent and annually adds 30,000–40,000 new cases [3,6]. Leishmaniasis is a disease characterized by high epidemiological dynamism related to constantly changing transmission conditions depending on the environment, demography, human behavior, socio-economic status and the human immune system [12,13,14]. *Leishmania infantum* is one of the most important *Leishmania* spp. worldwide from both a veterinary and a public health point of view. Domestic dogs (*Canis familiaris*) are believed to be the principal hosts of this protozoan and the main reservoirs of zoonotic human VL, which if left untreated is potentially lethal [15,16]. Today, sero-molecular diagnostic techniques have shown that the prevalence rates of Mediterranean canine leishmaniasis are much higher than previously assumed, probably due to the high percentage of asymptomatic infections (over 50%). [17]. However, other mammals infected with *L. infantum* have been reported in Italy [18,19]. The wide dispersal of VL indicates new reservoirs of the disease and the likely involvement of domestic animals, such as cats in the biological cycle of Leishmania. In Italy, *L. infantum* transmission is related to both the abundance and geographic distribution of competent vectors, mainly represented by *Phlebotomus perniciosus* [20,21,22]. *Phlebotomine* sandflies’ activity and their geographic distribution is influenced by different environmental parameters, such as land cover, altitude, air temperature, relative humidity and vegetation [23,24]. For example, air temperature is believed to condition the distribution of vector and seasonal sandfly activity [25]. Consistently, cases of canine and human Leishmaniasis have been historically described in central and southern regions of Italy, which are typically characterized by temperate winter and hot summers and [26,27], although in recent decades, numerous cases of animal and human Leishmaniasis have been reported in Northern Italian regions, traditionally classified as nonendemic areas for Leishmaniasis [27]. Although disease notification is required, available data are based on passive case detection. In some areas, the number of people and animals exposed to infection or asymptomatically infected is greater than the number of cases detected. Therefore, a One-Health strategy incorporating entomological, environmental animal and human data has been proposed as a valid alternative to study and control the Leishmaniasis transmission [28,29]. With this study, we evaluated, through a One-Health approach, the risk of transmission of *L. infantum* in the region of Sicily, an island in southern Italy known to be an endemic region for Leishmania, causing human, canine and feline Leishmaniasis [30,31,32,33]. Human and veterinary medicine, environmental sciences, and wildlife conservation specialists share a common interest in limiting visceral Leishmaniasis. However, the aforementioned disciplines respond to Leishmaniasis in separate ways. This retrospective study reports the results of 9 years (from 2013 to 2021) of surveillance based on integrated clinical and epidemiological sources of human and animal Leishmaniasis in Sicily with the aim to promote inter- and intra-sectoral collaboration to plan future control strategies.

## 2. Materials and Methods

### 2.1. Study Area

Sicily is an island in the south of Italy with a surface of 25.711 km^2^ (Longitude: 14.0153557, Latitude: 37.5999938, mean elevation: 622 m/2041 feet, Barometric Pressure: 94 Kpa). The island is predominantly mountainous, characterized by the presence of Mount Etna (10,900 feet, 3220 metres), with intense volcanic activity. The region of Sicily is divided into nine provinces: Palermo (PA), Trapani (TP) and Agrigento (AG) in the west, Caltanissetta (CL) and Enna (EN) in the center and Ragusa (RG), Siracusa (SR), Catania (CT) and Messina (ME) in the east.

### 2.2. Clinical Samples

Clinical samples from human patients were collected for diagnostic purposes and were examined at the dermatology and infectious diseases units of Sicilian province hospitals. VL patients presented such symptoms as: fever, hepatomegaly and/or splenomegaly; cutaneous lesions were erythematous papules, itchy, slow-growing papules that over a few weeks/months may progress into nodules and later to ulcers. Patients of VL were given treatment with liposomal amphotericin B (AmBisome^®^) 3 mg/kg administered intravenously (IV) once at days 1–5, 14, and 21. CL cases were treated with pentavalent antimonial (SbV) therapy, using a standard daily dose of 20 mg of SbV per kg, administered IV or via intramuscular route (IM). Overall, 341 Sera and 341 bone marrows samples were collected from humans with suspected VL; 699 tissue/biopsies were collected from humans with suspected CL, 74,349 lymph node needle aspirations and 74,349 sera were collected from dogs and 4774 lymph node needle aspirations and 4774 sera were collected from cats from veterinary medical centers (Table S1).

### 2.3. Diagnosis

All samples analyzed were from patients whose *Leishmania* infection status was screened for different reasons such as confirmation or exclusion of the disease, early diagnosis and investigation after therapy. Diagnosis of the disease was conducted by indirect immunofluorescence testing (IFAT) on serum matrices and detection of parasite DNA in bone marrow, tissue/biopsies and lymph node needle aspiration by real-time PCR (qPCR)). Serological examination included IFAT assays following the protocol according to the Office International des Epizooties (OIE) Terrestrial Manual (sensitivity 0.96, specificity 0.98) [34]. Sera were prepared by serial two-fold dilutions (1:40 to 1:5120 for canine samples; 1:80 to 1:5120 for human and feline samples) in phosphate buffered saline (PBS) at pH 7.2 and added to antigen coated wells. The IFAT slides were incubated for 30 min at 37 °C and positive and negative controls were included in each series of analyzed samples. The slides were subjected to three consecutive washes in PBS (10 min each), incubated with fluorescein labeled goat immunoglobulin (i.e., anti-cat, anti-dog and anti-human IgG, Sigma Aldrich, Saint Louis, MO, USA) and incubated at 37 °C for 30 min. The slides were washed three times (10 min each) in PBS and the sera reactivity was detected using a Leica DM 4000B fluorescence microscope (Leica, Heerbrugg, Switzerland, 40× magnification). Sera showed reactivity were subjected to analysis at dilutions 1:40 and 1:5120. The samples that showed titers of ≥1:160 were considered positive [34]. DNA was extracted from different clinical samples using PureLink™ Genomic DNA Mini Kit (Thermo Fisher Scientific K182002, Waltham, MA, USA) following the manufacturer’s instructions. The real-time PCR was performed out in a LightCycler^®^ 96 (Roche Life Science, Upper Bavaria, Germany) and carried as previously described (sensitivity 0.97, specificity 1) [35].

### 2.4. Entomological Survey

A phlebotomine sandfly survey was carried out to confirm the presence of *Leishmania* competent vectors and assess possible variations in their distribution in Sicilian provinces. CDC miniature light traps (Hausherr’s Machine Works, Toms River, NJ, USA) were used for 6 months (from the first week of May to the last week of October) of 2013 and 2021. Collected sandflies were stored in 70% ethanol and identified by light microscope using specific morphological keys [36,37].

### 2.5. Ethical Statement

Samples were collected as part of routine diagnosis and/or post-treatment follow-up without any necessary or additional noninvasive procedures and written informed consent was obtained from the patient at the time of the clinical examination. Patient records and information was anonymized and de-identified before analysis. All adult subjects provided written informed consent and a parent or guardian of any child participant provided written informed consent on their behalf. All animal samples were collected for diagnostic use and no unnecessary invasive procedures were performed, including parasitological confirmation of FeL or CanL. At the time of clinical examination, oral informed consent was obtained from the owners of dogs and cats.

### 2.6. Statistical Analysis

Data were analyzed using GraphPad Prism 9 software. Descriptive analysis of the qualitative variables was evaluated considering percentages, the number of cases and annual incidence of Leishmaniasis per 100,000 inhabitants. Chi-square and Fisher exact tests were conducted to compare proportions. The differences were considered statistically significant when *P*-value was ≤0.05. Posteriorly, maps representing positive cases of CL, VL, CanL and FeL were constructed. The results were graphically depicted on thematic maps stratified according to the following positivity parameters for CanL: low (0.02 to 7.22%), medium (7.23 to 16.29%), high (16.3 to 29.31%), intense (29.32 to 51.88%), and very intense (51.89 to 100%) [36]. The study was conducted by geographic information system (GIS) technology (MapInfo^®^ professional 17.0) software for generating the choroplethic maps. To estimate True Prevalence in dogs, cats and humans, the sensitivity and specificity estimates of each of qPCR and IFAT were imputed in the Rogan and Gladen formula: Tp = (Ap + CSps − 1)/(CSes + CSps − 1), where Tp = true prevalence; Ap = apparent prevalence; CSes = combined sensitivity of the test series (Se qPCR × Se IFAT), and CSps = combined specificity of the test series (1 − (1 − Sp qPCR) × (1 − Sp IFAT)) [38,39].

## 3. Results

### 3.1. Canine and Feline Data

**Figure 1:**
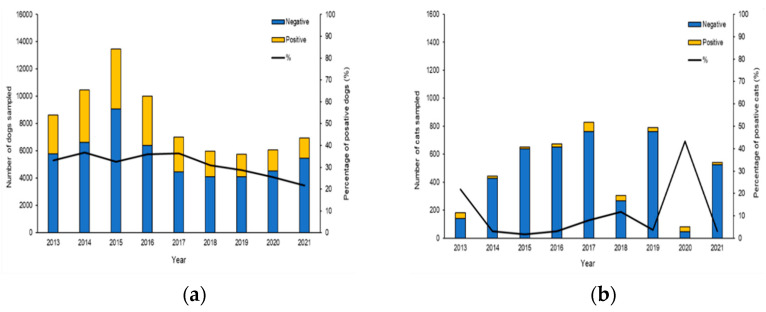
Annual trend and prevalence of CanL and FeL in Sicily, from 2013 to 2021. (**a**) Histogram showing the percentage of positive dogs between 2013 and 2021. (**b**) Histogram showing the percentage of positive cats between 2013 and 2021.

Investigations were initiated following the occurrence of autochthonous clinical cases reported by veterinarians, local medicals and confirmed by serological and molecular methods at The National Reference Center for Leishmaniasis (C.Re.Na.L). Analysis of epidemiological records from 2013 to 2021 with one or more diagnostic tests (Table S1) identified 74,349 tested dogs and 4774 tested cats living in Sicily (Figure 1). The results of our study revealed a true prevalence of 34.36% of CanL (adjusted Rogan-Gladen estimator) showing highest prevalence from 2013 to 2019, while in the years 2020 and 2021, we observed a slight decrease in prevalence with values of 25.4% and 21.6%, respectively (Figure 1a). The true prevalence of FeL obtained in our study period was 11.8% and the minimum number of feline samples was recorded in 2020 with 81, while the maximum number of samples was recorded in 2017 with 828 (Figure 1b).

**Figure 2:**
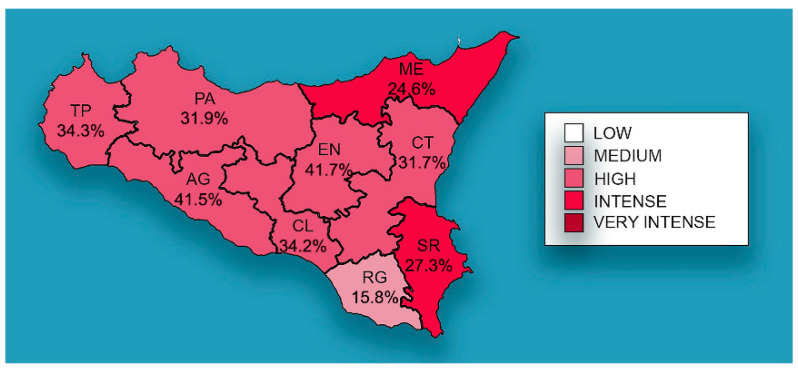
Mean prevalence of CanL by Sicilian province. The spatial distribution of mean prevalence (%) from 2013 to 2021. The map generated with geographic information system (GIS) technology (MapInfo^®^ professional 17.0) software.

The distribution of mean prevalence of CanL in the Sicilian provinces is shown in Figure 2, with EN (41.7%) and AG (41.5%) recording the highest prevalence, followed by TP (34.3%), CL (34.2%), PA (31.9%), CT (31.7%), SR (27.3%) ME (24.6%), and RG (15.8%).

### 3.2. Human Data

**Figure 3:**
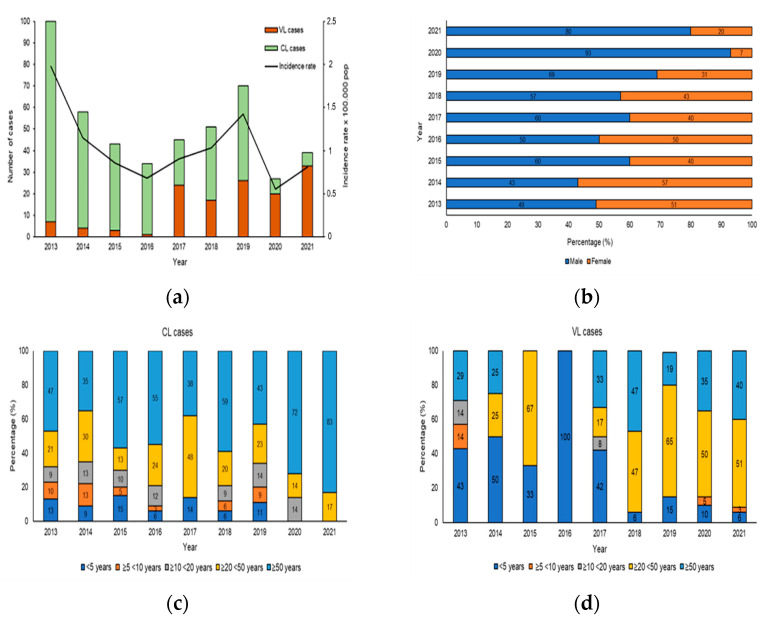
Human Leishmaniasis in Sicily. (**a**) Number of patients and incidence rates of cutaneous and visceral Leishmaniasis in Sicily, from 2013 to 2021. The histogram shows the incidence rates of CL and VL cases (per 100,000 pop) between 2013 and 2021 (Source: ISTAT). (**b**) Proportion of cutaneous and visceral Leishmaniasis by sex from 2013 to 2021, *p*-value < 0.0001. (**c**) Proportion of CL by age group from 2013 to 2021. (**d**) Proportion of VL cases by age group, *p*-value < 0.0001.

From 2013 to 2021 in Sicily, 467/1040 (true prevalence 48.2%) samples were positive for human Leishmaniasis, including 332 for CL and 135 for VL and the results distribution of molecular and serological analysis were shown in Table S1. The analysis of human Leishmaniasis included temporal distribution, sex and age. Figure 3a shows the evolution of the number of VL/CL cases and incidence rate reported in the study period from 2013 to 2021. The increase in the number of cases of CL and VL reported in 2013 and 2019 was responsible for the peaks observed in the overall Leishmaniasis case count in Figure 3a. During the study period, the cumulative incidence was 1.04 per 100,000 populations, with the highest incidence observed in 2013 (1.9 per 100,000 population) and the lowest in 2020 (0.5 per 100,000 population). During the first 4 years of the study, a higher number of CL cases was observed, whereas in the second period (2017–2021), there was a decrease in CL and an increase in VL cases. The distribution of CL and VL cases according to sex is shown in Figure 3b. The affected population consisted of 275 males (61.7%) and 192 females (38.3%), indicating an important and statistically significant sex bias (χ^2^ = 35.48, df = 8, *p* < 0.001). To reveal the association of CL and VL with age during the 9 years of the study, the data were stratified according to 5 age groups: <5 years, from 5 to 9 years, from 10 to 19 years, from 20 to 49 years and ≥50 years of age. While all age groups were affected by CL (Figure 3c), we observed significant, age-specific differences (χ^2^ = 169, df = 32, *p* < 0.001), with the highest CL prevalence seen in adults ≥20 < 50 (23.3%) and ≥50 years of age (54.3%). Likewise, statistically significant, age-specific differences were revealed for VL (χ^2^= 607, df = 32, *p* < 0.001), which mainly affected the age group ≥20 < 50 years between 2018–2021 (54.2%), while between 2013–2017, 66.6% patients were aged ≤5 years (Figure 3d).

**Figure 4:**
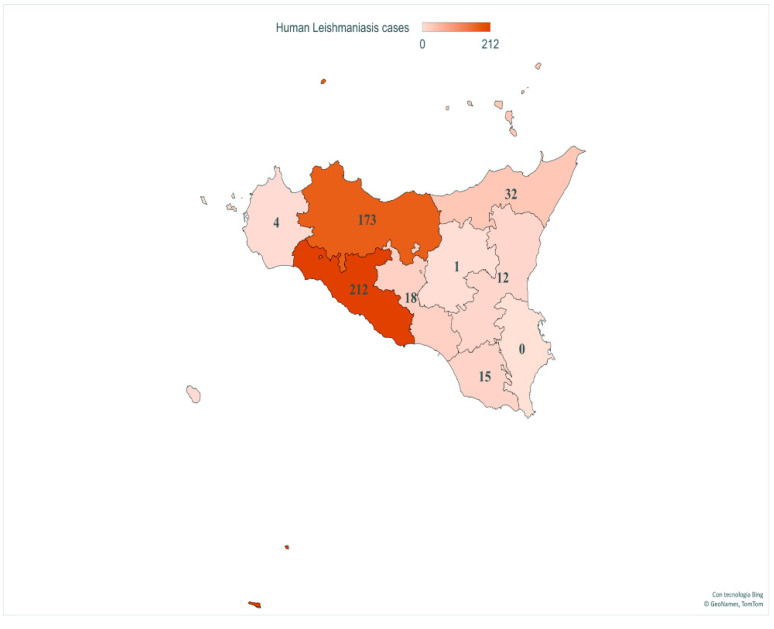
Number of VL and CL patients in provinces of Sicily. This image presents the spatial distribution of human case counts from 2013 to 2021. Map generated with Excel Power Map.

Finally, geospatial localization revealed CL and VL cases in all provinces of Sicily. The provinces with the highest number of patients were AG (212/467, 45.4%) and PA (173/467, 37%), followed by ME (32/467, 6.8%), CL (18/467, 3.85%), RG (15/467, 3.21%), CT (12/467, 2.56%), TP (4/467, 0.85%) and EN (1/467, 0.21%) (Figure 4).

### 3.3. Sandfly Data

**Figure 5:**
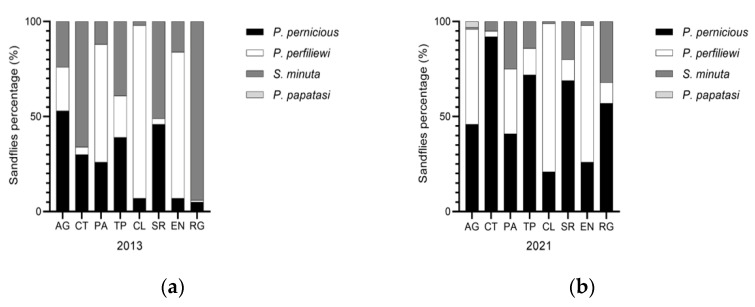
Proportion of sandfly species by Sicilian provinces. (**a**) Phlebotomus species collected during 2013 year; and (**b**) Phlebotomus species collected during 2021 year.

During 2013 in Sicily, 21,121 Phlebotomus were captured, identifying 3 species: *P. perniciosus, P. perfiliewi, S. minuta. P. perfiliewi* was the most abundant (68.7%), followed by *P. perniciosus* (17.2%) and *S. minuta* (14%), showing *P. perfiliewi* was the dominant species in Sicily [40,41]. In 2021, 21,716 *Phlebotomus* were captured identifying 4 species: *P. perniciosus, P. perfiliewi, S. minuta and P. papatasi. P. perfiliewi* was confirmed as the most common species in Sicily (61.6%), followed by *perniciosus* (with a percentage increased to 33.1%), and a reduction in *S. minuta* (4.7%). During the entomological analysis in 2021, an interesting finding was the identification of 90/21716 (0.41%) specimens of *P. papatasi* in the AG province (Table 1) and the proportion of sandfly species by Sicilian provinces was shown in Figure 5.

## 4. Discussion

One-Health is a concept rooted in the history of medicine and healthcare. It proposes a holistic view of the distinct disciplines of veterinary medicine, human medicine, wildlife conservation and environmental science [42,43]. This concept is especially important for vector-borne zoonoses, such as Leishmaniasis. The dynamics and temporal–spatial evolution of Leishmaniasis in Europe depends on various factors, including global warming that affects the ecology and distribution of phlebotomine vectors, and anthropogenic risk factors such as migration, travel, animal trade and environmental modifications [44]. The region of Sicily, an island in southern Italy, is considered endemic for *L. infantum* [31,33,45,46]. With the current study, we used a One-Health approach, integrating human and animal data, to assess and map the risk of endemic transmission of *Leishmania* in region Sicily, covering a time span of 9 years. We found positive associations between human cases, infected reservoir hosts and vector spatial distribution. Our study revealed a mean true prevalence of 34.4% of CanL during the years under investigation, confirming Leishmaniosis as an endemic public health threat in Sicily [33,45]. The highest prevalence of CanL in Sicily was recorded from 2013 to 2019, since the sampling of dogs included in the study was carried out mainly for diagnostic purposes, following a clinical suspicion. From 2020, the establishment of the “Regional monitoring plan for Leishmaniasis in the territory of the region of Sicily” (decree 473/2020), which required an active surveillance of dogs in health shelters and public kennels, resulted in a slight decrease in prevalence in the years 2020 and 2021 with values of 25.4% and 21.6%, respectively. 

Diagnosis based only on the presence of clinical signs related to the disease could overestimate the prevalence of leishmaniasis in this area, and since the data were collected only in the context of routine diagnosis, following a diagnostic suspicion, the data obtained certainly led to a selection BIAS, since the selection of individuals for analysis was not randomized. The risk of collecting biased samples that do not represent the entire population represents a limitation. This study therefore underlined the importance of a surveillance plan by random sampling, as it allows (i) identification of asymptomatic carriers in endemic areas, (ii) valid estimation of the disease prevalence, and (iii) control programs for the prevention and treatment of disease.

The sensitivity and specificity of IFAT and qPCR are singularly high and close to 100%, but the use of an integrated diagnostic approach of these techniques can try to solve the diagnostic problems the low predictive values of serology, whose results can be affected by either persistent antibodies (false positive) or immunosuppression (false negative) and the inability to detect parasitic DNA in some samples, thus considerably increasing sensitivity and specificity. The prevalence of CanL in the Sicilian provinces showed a homogeneous distribution pattern in all provinces, with the exception of EN and AG, although the data for the province of EN is due to a low sample number, overestimating disease prevalence. Unlike dogs, cats have been considered as incidental hosts resistant to Leishmaniasis for many years. However, this feline now appears to be an important part of the ecological system in which Leishmania parasites can persist indefinitely [47]. Several studies on Leishmania infection in cats have been conducted in recent years [48]. Feline Leishmaniasis has gained importance, being considered today as an emerging disease. Recent epidemiological studies suggest that cats living in Leishmania endemic areas have similar prevalence data and exposure risk as dogs in the same area, although they are generally subclinical or asymptomatic [46,49]. When associated with disease, FeL can include a variety of signs, with skin or muco-cutaneous lesions and enlarged lymph nodes are being the most common [16,49]. Indeed, dermatologic lesions predominate in the clinical presentation of FeL, representing more than half of the clinical manifestations [16,49]. From 2013 to 2021, the true prevalence of FeL obtained in our study in Sicily was 11.8%. It is important to discuss some potential limitations of the data obtained: such as the low sample number and an unequal distribution of cases in the Sicilian provinces, as the cats are primarily from Messina and Palermo provinces. However, our study may suffer from underestimation. First, immunopathogenesis of FeL is poorly understood. This protozoonosis shows a wide range of clinical signs and clinicopathologic abnormalities, which coupled with the lack of standardized protocols, make diagnosis even more difficult for veterinarians [48]. Second, the diagnosis of FeL is usually based on the results of histopathology, cytology, immunohistochemistry, culture, PCR and serology. In addition to the inherent limitations and advantages of each of these methods, their diagnostic value depends on many conditions, including the reagents, the biological sample used and the particular technique used. The most common laboratory method for diagnosing FeL is the IFA test [47]. However, care should be taken before confirming FeL with IFAT; in fact, many present with clinical symptoms of Leishmaniasis, but are negative on IFAT and should be subjected to other additional diagnostic tools such as cytology and PCR [47]. Our surveillance was not limited to canine and feline Leishmaniosis, but it extends to infections of human patients showing clinical signs of VL and CL. The cumulative incidence during these 9 years of observation was 1.04 per 100,000 inhabitants, with the highest incidence found in 2013 (1.9 per 100,000 inhabitants) and the lowest in 2020 (0.5 per 100,000 inhabitants). During the study period, a change in the type of Leishmaniasis was observed with an increase in CL cases from 2013 to 2019, and an increase in VL cases from 2020 to 2021, probably due to the coronavirus pandemic in Italy changing the way of practicing dermatology and because most of the departments are closed. Between 1989 and 2009, Italy reported an increase in the number of VL cases from a baseline of about 10–30 cases annually notified since the 1950s [50]. However, there is an increasing trend in Italy, which could be associated with two factors: first, the reporting performance of the VL system in Italy, although acceptable, varies from region to region, while CL is certainly under-notified nationally because it often does not necessitate hospitalization; and second, several health centers specializing in infectious diseases are available regionally, consequently the diagnosis of Leishmaniasis is not centralized. Thus, the knowledge about the epidemiology of *L. infantum* is still insufficient, and our surveillance data in the Sicily region are discrepant with those in Italy, which may be affected by underestimation. Therefore, C.Re.Na.L. in a One-Health perspective, supporting the diagnosis of the main Sicilian nosocomial showed a high incidence of human Leishmaniasis in Sicily. Our study has shown that the percentage of affected men in Sicily was higher as compared to women in Sicily, from 2013 to 2021. Recent studies have described a male bias in certain infectious diseases [51], including Leishmaniasis [52], suggesting behavioral and physiological theories. However, we cannot exclude that men are more exposed to infected vectors because of their social or working activities [53]. By age groups in individuals with CL, our results obtained found the support of another study that examined epidemiological aspects of CL by age, observing higher Leishmaniasis infection in people older than 50 years of age and correlating it with occupation and increased outdoor living [52]. VL patients were in the largest percentage in the age group ≥20 < 50 years from 2018 to 2021, while from 2013 to 2017, the age group ≤5 was the largest. In the younger population, the main causes are related with a lack of an efficient immune response [51,53]. This is in line with findings in other similar studies [54,55]. In adults, the disease is associated with states of immunosuppression, due to treatment or diseases such as HIV, whereas in children it could be due to lack of an effective immune response [56]. According to our data, 467 people have been infected with Leishmania in the past 9 years, and considering the geospatial location of infected humans, CL and VL cases were observed in all provinces of the island except SR (Figure 4). The reasons for this absence and/or reduced number of cases are unclear, but may be related to a lack of a surveillance system, a diagnosis system, and a possible underestimation of clinical suspicion at the primary care level. We found positive associations between VL and CL cases and the geographic distribution of CanL, in the provinces of AG and PA, indicating that strains of *L. infantum* were circulating in some areas of the region during the study period. While CanL, FeL, CL, and VL cases referred to a time span up to 2013 to 2021, the entomological samples were based on monitoring activities carried out only in 2013 and 2021. Our entomological results supported that *P. perfiliewi* represented the principal vector of *L. infantum* in some areas of VL endemicity in the Mediterranean basin according to available entomological investigations on *L. infantum* vectors in southern Sicily [57,58]. The presence of *P. papatasi* on the island was to be considered non-threatening to people and dogs, given its specificity in transmitting *Leishmania major* [59]. *L. major* caused CL, is endemic in Africa, thus potentially importable by the migration phenomenon; however, the risk of local transmission was reduced due to the absence in Sicily of gerbils, natural reservoir hosts [45]. However, the fact that *Leishmania spp.* can undergo genetic exchange by forming *L.* *major/L. infantum* hybrids, efficiently transmitted by the specific vector *P. papatasi*, could result in increased transmission potential [59,60]. These data highlight the potential epidemiological impact of genetic switch in parasites; in fact, hybrids may have the potential risk to expand disease transmission and invade new territories [59]. This suggests that in the wild and especially in Sicily, hybrid strains may spread using *P. papatasi* sandfly vectors, thus increasing the risk of their spreading into new foci. The presence of *P. papatasi* in AG could be correlated with the high number (212/467, 45.4%) of human cases found in this province, but further investigation is needed. Regarding the distribution of phlebotomine species in the provinces of the island, it was found that the species *S. minuta* in 2013 was predominantly found in the eastern part of Sicily (RG, CT, SR and EN), while in 2021, the distribution remains the same although with a significant reduction. Climate change is allegedly affecting the seasonality of many species, including several insect vectors, geographic distribution and increased connectivity has facilitated the expansion of sandflies into new geographic areas [7,61]. Even though in Sicily Leishmaniasis is endemic, this region will continue to be a highly transited island, with international tourism, a higher flow of migration, that can facilitate the occurrence of imported cases. Thus, trying to work on a One-Health concept that integrates the different fields involved in transmission processes, including entomological, animal, and human data, is of fundamental importance to know the risk of transmission and spread of the parasite. The One-Health approach is the perfect concept to apply on studying Leishmaniasis by a holistic view of the previously various professions belonging to human and veterinary medicine, environmental science and wildlife conservation. It is a parasitosis of great human and veterinary medical relevance that involves a complicated interaction between a pathogenic protozoan, sandflies vectors, environmental influence on vector distribution, a small reservoir of companion animal (dog) infection, and susceptible human populations. An interdisciplinary team of human physicians, microbiologists, veterinarians, parasitologists, entomologists, ecologists, epidemiologists, immunologists, and public health officials are essential for effective control against Leishmaniasis. In addition, the One Health approach is well suited to the surveillance and control requirements of this infection. This study examines the current state of Leishmaniasis prevention, control, and surveillance in Sicily, but it also reveals gaps in the islands under study that could be remedied through the application of the One-Health principles we have been discussing.

## Figures and Tables

**Table 1:** *Phlebotomine* sandfly species recorded in Sicilian provinces during 2013 and 2021.

**Specimens of *Phlebotomus* Species Collected in 2013**					
**Provinces**	***P. pernicious***	***P. perfiliewi***	***S. minuta***	***P. papatasi***	**Total**
Agrigento	1593	683	703	0	2979
Catania	312	36	683	0	1031
Palermo	648	1572	315	0	2535
Trapani	43	24	43	0	110
Caltanissetta	664	8497	205	0	9366
Siracusa	47	3	53	0	103
Enna	325	3706	798	0	4829
Ragusa	8	2	158	0	168
Total (%)	3640 (17.2)	14,523 (68.7)	2958 (14)	0	21,121
**Specimens of *Phlebotomus* Species Collected in 2021**					
**Provinces**	***P. pernicious***	***P. perfiliewi***	***S. minuta***	***P. papatasi***	**Total**
Agrigento	1384	1505	30	90	3009
Catania	1205	39	66	0	1310
Palermo	1039	861	633	0	2533
Trapani	86	17	17	0	120
Caltanissetta	1987	7379	94	0	9460
Siracusa	142	23	41	0	206
Enna	1281	3548	99	0	4928
Ragusa	85	17	48	0	150
Total (%)	7209 (33.2)	13,389 (61.7)	1028 (4.7)	90 (0.4)	21,716

## Data Availability

Not applicable.

## Supplementary Materials

The following supporting information can be downloaded at: https://www.mdpi.com/article/10.3390/microorganisms10091704/s1, Table S1: Results distribution of molecular and serological analysis in humans, canines and felines population.
