# Supplementary material for: Retrospective Analysis of Leishmaniasis in Sicily (Italy) from 2013 to 2021: One-Health Impact and Future Control Strategies
Source: Microorganisms. 2022 Aug 24;10(9):1704. doi: 10.3390/microorganisms10091704 (PMC9504907; doi:10.3390/microorganisms10091704)
Supplement: Supplementary file 1 [file microorganisms-10-01704-s001.zip › microorganisms-1870395-supplementary.pdf]

Table S1. Results distribution of molecular and serological analysis in humans, canines and felines population.

Species	Clinical Presentation (number)	Clinical samples (number)	IFAT[1] Positive/total	qPCR[2] Positive/total	Positive patients selection
Humans	VL[3] (135)	Serum (341)	100/341	n.a.[4]	- 110 VL qPCR positive, IFAT positive; - 25 VL qPCR positive, IFAT negative;
		Bone marrow (341)	n.a.	135/341	
	CL[5] (332)	Tissue/skin biopsy (699)	n.a.	322/699	- 322 CL qPCR positive;
Canine	CanL[6] (23794)	Serum (74349)	23794/74349	n.a.	-22755 CanL qPCR positive, IFAT positive; - 1035 CanL IFAT positive, qPCR negative; - 4 CanL qPCR positive, IFAT negative;
		Popliteal lymphnode (74349)	n.a.	23794/74349	
Feline	FeL[7] (274)	Serum (4774)	109/4774	n.a.	-109 FeL qPCR positive, IFAT positive; - 165 FeL qPCR positive, IFAT negative;
		Popliteal lymph node (4774)	n.a.	274/4774	

[1]IFAT, indirect immunofluorescent assay; [2]qPCR, quantitative PCR; [3]VL, visceral leishmaniasis; [4]n.a., not applicable; [5]CL, cutaneous leishmaniasis; [6]CanL, canine leishmaniasis; [7]FeL, feline leishmaniasis.
